# Large language model evaluation and feedback of transcribed simulated physician-patient verbal interactions

**DOI:** 10.3389/frai.2026.1936749

**Published:** 2026-09-21

**Authors:** Eric J. Nolan, Harry B. Burke

**Affiliations:** 1 Division of Hospital Medicine, Department of Internal Medicine, College of Medicine, The Ohio State University Wexner Medical Center, Columbus, OH, United States; 2 George Washington University School of Medicine, Washington, DC, United States

**Keywords:** clinical communication, generative artificial intelligence, large language models (LLMs), narrative feedback, physician patient interaction

## Abstract

**Introduction:**

Physician–patient verbal interactions are fundamental to clinical care and medical education, yet their evaluation is time-intensive and subject to inter-rater variability. We evaluated whether large language models (LLMs) can accurately assess transcribed standardized patient-physician verbal interactions and generate useful narrative feedback.

**Methods:**

In this single-center study, 12 standardized patient–medical student encounters were independently evaluated by ChatGPT-4o, Gemini 3 Pro, and a physician reviewer using a gold standard rubric composed of 55 history-taking elements and 19 presentation elements assessing diagnostic reasoning, management planning, and patient counseling. Agreement was assessed using Cohen’s kappa and intraclass correlation coefficients. Narrative feedback generated by the LLMs was independently evaluated by ChatGPT, Gemini, Claude, and a physician reviewer.

**Results:**

Across raters, mean scores were similar, with 35–36 of 55 history-taking elements and 13–14 of 19 presentation elements identified as present. Agreement was high for history-taking elements (*κ* = 0.84–0.89; intraclass correlation coefficients = 0.93–0.97) and lower for presentation elements (*κ* = 0.62–0.72; intraclass correlation coefficients = 0.61–0.83). Both LLMs generated detailed, accurate, and clinically useful narrative feedback.

**Discussion:**

These findings demonstrate that LLMs can reliably evaluate simulated physician–patient interactions and may support standardized assessment of clinical communication for research, quality improvement, and medical education.

## Background and significance

Physician-patient verbal interactions are fundamental to clinical medicine. During these encounters, clinicians obtain diagnostic information, communicate diagnoses and treatment plans, engage patients in shared decision-making, and provide emotional support (Burke et al., 2014, 2015). Effective communication has been associated with improved patient satisfaction, adherence to treatment, and clinical outcomes, whereas poor communication has been linked to medical errors, reduced adherence, malpractice claims, and increased healthcare utilization (Chen et al., 2025; Lazris et al., 2021; Navarro et al., 2021; Zolnierek and DiMatteo, 2009; Levinson et al., 1997). Assessment of physician-patient interactions is therefore important for clinical practice.

Evaluation of physician-patient interactions (PPIs) can identify strengths and deficiencies in communication, guide targeted feedback, and improve clinician performance (Gilligan et al., 2021; Lai et al., 2020; Drossman et al., 2021). Traditionally, such assessments have relied on trained human raters. Although human evaluation remains the standard approach, it is resource intensive and may be influenced by fatigue, inconsistency, and subjective interpretation (Hillen et al., 2025; Scarff et al., 2023; Hultman et al., 2019; Kaye et al., 2018; Brannick et al., 2011; McLaughlin et al., 2009; Harasym et al., 2008; Landon et al., 2003). Furthermore, meaningful comparisons across encounters can be difficult because physician–patient interactions vary substantially according to clinical context, patient characteristics, and clinician experience.

Recent advances in generative artificial intelligence have led to an interest in the use of large language models (LLMs) for assessment of clinical performance (Hui et al., 2026; Thirunavukarasu et al., 2023). Prior studies have demonstrated that LLMs can evaluate written clinical documentation, generate educational feedback, assess communication quality, and analyze physician–patient conversations (Burke et al., 2024; Fenton et al., 2025; Zhou et al., 2025; Runyon, 2025; Wang et al., 2024; Herschbach et al., 2025).

Despite increasing interest in LLM-based assessment, relatively little research has examined the standardized evaluation of physician–patient verbal interactions. We recently demonstrated that LLMs can accurately transcribe standardized patient–medical student interactions (Nolan and Burke, 2026). Evaluating physician–patient verbal interactions is challenging because clinical encounters vary substantially across medical conditions, patients, and clinicians. To provide a standardized and controlled assessment environment, we used a standardized patient–medical student-as-physician paradigm. Building on our prior work, the present study evaluates whether LLMs can accurately score transcribed verbal interactions using a predefined gold standard rubric and generate useful narrative feedback. We hypothesized that ChatGPT and Gemini will demonstrate high agreement with human raters and provide clinically useful feedback.

## Methods

### Study design and setting

This retrospective single-center study analyzed standardized patient (SP)-medical student verbal interactions conducted as part of a clinical skills assessment. The study was reviewed by the Institutional Review Board and determined to be exempt.

### Standardized patient encounters

A predefined gold standard chest pain case was selected because it represents a common and clinically important presentation. During each encounter, third-year medical students obtained a focused history, performed a physical examination, and presented their assessment, management plan, and patient counseling to the patient.

SPs were trained on the case and instructed to portray the patient consistently across encounters. Eight SPs participated in 52 recorded encounters. To create a representative sample while maintaining feasibility, the three SPs with the largest number of encounters were identified, and four encounters from each SP were randomly selected, yielding 12 encounters for analysis.

### Audio recording and transcript preparation

Audiovisual recordings took place in carpeted 8 × 10 ft. examination rooms with 8-ft ceilings using a centrally mounted Parlé TCM-1 ceiling microphone (Biamp, Beaverton, Oregon, United States) with default settings. The interactions were recorded using SimCapture (Laerdal Medical, Stavanger, Norway). Recordings were downloaded to a PC in.m4v format, and Audacity (Muse Group, Limassol, Cyprus) was used to extract the audio as.wav files. The audio recordings were not altered, muted, clipped, or otherwise modified. The audio files were manually transcribed by a physician to produce gold-standard transcripts. Metadata associated with the audiovisual recordings, extracted audio files, and transcripts did not contain student names or other identifying information. Each recording was reviewed at least three times to ensure completeness and accuracy, and a second physician independently verified all transcripts.

### Gold standard rubrics

The gold standard rubric assessed whether specific items of information (“elements”) were obtained and communicated during the encounter. These elements were based on the information that students were expected to elicit and communicate during the encounter. The evaluation consisted of two components: history taking (history-taking component) and the formulation and presentation of the assessment and plan (presentation component). The history-taking rubric included 55 elements organized into six categories: chief complaint (2 elements), history of present illness (16 elements), review of systems (11 elements), past medical history/medications/allergies (13 elements), family history (3 elements), and social history (10 elements) (Supplementary Table S1). The presentation rubric included 19 elements organized into three categories: assessment (6 elements), plan (10 elements), and communication and closing (3 elements) (Supplementary Table S2). Each element was assessed as either present or absent. History-taking elements were scored as present if the student elicited the specified information and absent if the information was not obtained. Presentation elements were scored as present if the student communicated the specified information to the patient and absent otherwise. Present elements were considered correct and absent elements incorrect.

Standardized-patient errors were uncommon, averaging one error per encounter. When an SP provided incorrect information, the affected statement was removed from both the transcript and the corresponding element item before scoring.

### Raters

Three raters independently evaluated each encounter: ChatGPT-4o (OpenAI, San Francisco, California, United States), Gemini 3 Pro (Google, Mountain View, California, United States, and a human physician reviewer. The LLMs were used through their standard consumer interfaces; model parameters (e.g., temperature and top-p) were not user-specified. Memory and reference to prior chats were disabled. Each encounter was evaluated in a separate new chat session, such that the model had no conversational context from previously evaluated encounters.

### Evaluation procedure

Student evaluation was conducted in two phases.

In Phase I, raters assessed whether 55 predefined elements of history-taking were elicited during the encounter. The raters were given a prompt (Figure 1a) and a rubric with the list of history-taking elements (Supplementary Table S1). Each history-taking element was classified as present or absent, and the total number of correct elements was calculated.

**Figure 1:**
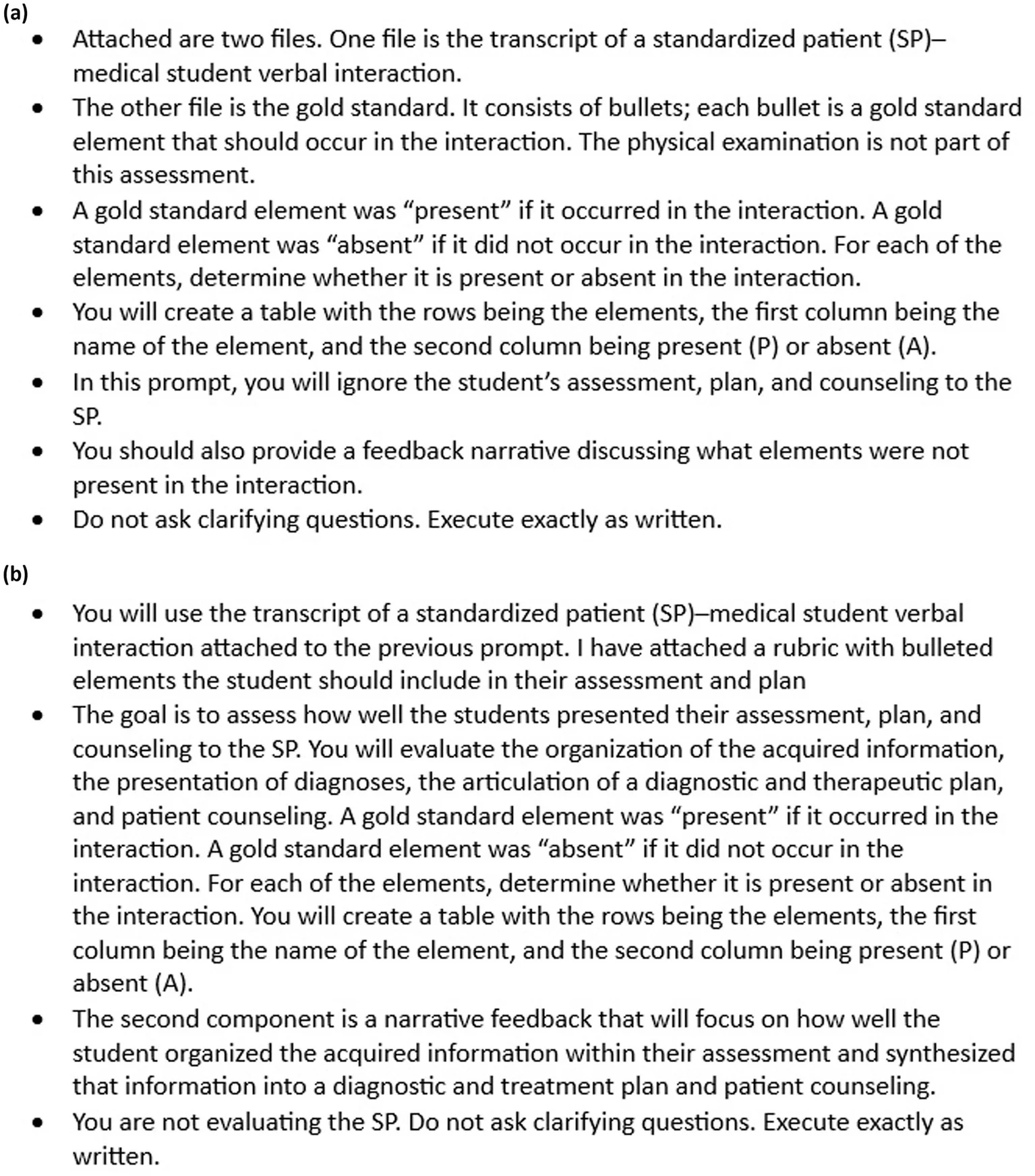
Rater prompts used in Phase I **(a)** and Phase II **(b)**.

In Phase II, raters assessed whether 19 predefined elements of presentation were present during the assessment, plan, and counseling portions of the encounter. The raters were given a prompt (Figure 1b) and a rubric with the list of presentation elements (Supplementary Table S2). Each presentation element was classified as present or absent, and the total number of correctly communicated elements was calculated.

For both phases, ChatGPT and Gemini also generated qualitative narrative feedback assessing strengths, deficiencies, and opportunities for improvement for the students.

All LLM evaluations were completed successfully. Neither ChatGPT nor Gemini produced incomplete responses, nonbinary classifications, or system errors requiring adjudication, exclusion, or repeat analysis. All elements received a present/absent rating, and narrative feedback was generated for all encounters.

### Statistical analysis

Performance was summarized as the mean number and percentage of elements scored as correct.

Inter-rater agreement for binary element-level ratings was evaluated using Cohen’s kappa (κ). Agreement was defined as both raters assigning the same score (present or absent) to a given element for a given student. Analyses of history taking elements included 660 element-level ratings per rater (55 elements × 12 students), resulting in 1,980 pairwise comparisons across the three rater pairs. Analyses of presentation elements included 228 ratings per rater (19 elements × 12 students), resulting in 684 pairwise comparisons. Cohen’s kappa was calculated from the complete set of element-level ratings by tabulating the number of elements scored as present by both raters, absent by both raters, present by one rater and absent by the other, or vice versa. No partial-credit or intermediate scoring categories were used; all elements were scored dichotomously as present or absent.

For each encounter, the proportion of elements scored as present by each rater was calculated separately for the history-taking and presentation components (Supplementary Table S3). Agreement in these encounter-level summary scores was assessed using intraclass correlation coefficients (ICCs) derived from a two-way random-effects model.

Interpretation of Cohen’s kappa followed the criteria of Landis and Koch, with values of 0.41–0.60 indicating moderate agreement, 0.61–0.80 substantial agreement, and >0.80 almost perfect agreement (Landis, 1977). ICC values were interpreted according to published guidelines, with values <0.50 considered poor, 0.50–0.75 moderate, 0.75–0.90 good, and >0.90 excellent agreement (Koo and Li, 2016).

To assess the potential impact of the small sample size and the influence of individual student-SP encounters and SPs, leave-one-out sensitivity analyses were performed. To evaluate the influence of individual students, each student-SP encounter was sequentially excluded, and Cohen’s kappa and ICC statistics were recalculated for all three rater pairs, yielding 12 leave-one-out analyses. To evaluate the influence of individual SPs, analyses were repeated after excluding all encounters associated with a given SP (four encounters per SP), yielding three additional analyses, each based on eight student-SP encounters. Sensitivity analyses were conducted separately for the history-taking and presentation components. The stability of agreement estimates and any changes in the qualitative interpretation of agreement were assessed.

### Evaluation of narrative feedback

Narrative feedback generated by ChatGPT and Gemini was independently reviewed by ChatGPT-4o, Gemini 3 Pro, Claude Sonnet 5 (Anthropic, San Francisco, California, United States), and a human evaluator. The LLM reviewers were used with default settings through their standard web interfaces and did not have access to prior conversations. Reviewers were blinded to the evaluations generated by the other reviewers. For each SP-student interaction, the narrative feedback generated by ChatGPT and Gemini was de-identified and presented together in a single document, with the source of each response labeled as Rater 1 or Rater 2. Reviewers were given the following prompt: “The attached documents include feedback from 2 different raters to 12 medical students who did the same OSCE case with a standardized patient. Compare the feedback between the raters.” The resulting reviewer assessments were evaluated and summarized with respect to accuracy, completeness, and usefulness of the rater feedback.

## Results

### Student performance

Across the 55 history-taking elements, mean (SD) scores were similar among raters: human reviewer, 36 ± 5 (67%); ChatGPT, 35 ± 5 (65%); and Gemini, 36 ± 5 (66%) (Table 1).

**Table 1:** Mean number of elements correct.

Agreement components	Human rater (mean, SD, %)	ChatGPT (mean, SD, %)	Gemini (mean, SD, %)
History-taking elements (*N* = 55)	36 (5), 67%	35 (5), 65%	36 (5), 66%
Presentation elements (*N* = 19)	13 (2), 69%	14 (2), 74%	14 (2), 73%

For the 19 presentation elements, mean (SD) scores were likewise comparable: human reviewer, 13 ± 2 (69%); ChatGPT, 14 ± 2 (74%); and Gemini, 14 ± 2 (73%) (Table 1). Overall, students correctly completed approximately two-thirds of the history-taking elements and nearly three-fourths of the presentation elements.

### Agreement for history-taking elements

Agreement for history-taking element scoring was high across all rater pairs. Among the 660 history-taking element ratings per rater, Cohen’s kappa was 0.84 (95% CI, 0.80–0.89) for ChatGPT–Gemini, 0.89 (95% CI, 0.86–0.93) for ChatGPT–human, and 0.89 (95% CI, 0.86–0.93) for Gemini–human (Table 2). According to standard interpretive guidelines, these values indicate close to perfect agreement.

**Table 2:** Agreement for history-taking elements.

Agreement measure	ChatGPT–gemini(mean, 95% CI)	ChatGPT–human (mean, 95% CI)	Gemini–human (mean, 95% CI)
Cohen’s kappa	0.84 (0.80–0.89)	0.89 (0.86–0.93)	0.89 (0.86–0.93)
ICC	0.97 (0.89–0.98)	0.94 (0.82–0.98)	0.93 (0.78–0.98)

Agreement in overall history-taking scores was similarly strong. ICC values were 0.97 (95% CI, 0.89–0.98) for ChatGPT–Gemini, 0.94 (95% CI, 0.82–0.98) for ChatGPT–human, and 0.93 (95% CI, 0.78–0.98) for Gemini–human (Table 2), demonstrating excellent reliability.

### Agreement for presentation elements

Agreement was lower for presentation-element scoring than for history-taking element scoring. Across the 228 presentation-element ratings per rater, Cohen’s kappa was 0.72 (95% CI, 0.62–0.82) for ChatGPT–Gemini, 0.62 (95% CI, 0.51–0.74) for ChatGPT–human, and 0.64 (95% CI, 0.53–0.75) for Gemini–human (Table 3). These values indicate substantial agreement.

**Table 3:** Agreement for presentation elements.

Agreement measure	ChatGPT–gemini(mean, 95% CI)	ChatGPT–human (mean, 95% CI)	Gemini–human (mean, 95% CI)
Cohen’s kappa	0.72 (0.62–0.82)	0.62 (0.51–0.74)	0.64 (0.53–0.75)
ICC	0.74 (0.34–0.92)	0.61 (0.13–0.86)	0.83 (0.47–0.94)

Agreement in overall presentation scores ranged from moderate to good. ICC values were 0.74 (95% CI, 0.34–0.92) for ChatGPT–Gemini, 0.61 (95% CI, 0.13–0.86) for ChatGPT–human, and 0.83 (95% CI, 0.47–0.94) for Gemini–human (Table 3). Confidence intervals were wider than those observed for history-taking element scoring, indicating reduced precision.

### Sensitivity analysis

Cohen’s kappa and ICC estimates for history-taking element agreement remained stable across all rater pairs in the leave-one-out sensitivity analyses (Supplementary Tables S4, S5). Cohen’s kappa values consistently indicated close to perfect agreement (0.82–0.94), and ICCs remained excellent (0.89–0.98), demonstrating that no individual encounter or SP materially influenced results.

Greater variability was observed in the leave-one-out analyses of presentation-element rating Cohen’s kappa and ICC. Following exclusion of individual student-SP encounters, Cohen’s kappa values ranged from 0.60–0.67 for Human-ChatGPT, 0.62–0.65 for Human-Gemini, and 0.70–0.77 for ChatGPT-Gemini comparisons; exclusion of individual SPs yielded similar results, with kappa values ranging from 0.55–0.74 across rater pairs (Supplementary Table S6). Following exclusion of individual student-SP encounters, presentation-element ICCs ranged from 0.57–0.86 for ChatGPT-Gemini, 0.41–0.70 for ChatGPT-Human, and 0.77–0.87 for Gemini-Human comparisons; exclusion of individual SPs produced similar findings (Supplementary Table S7). Despite this variability, the overall interpretation remained unchanged, with presentation-element agreement consistently lower than history-taking element agreement and remaining within the moderate-to-good reliability range.

Overall, the findings were robust to exclusion of individual encounters and SPs. Presentation-element agreement was more variable, likely reflecting the greater subjectivity involved in assessing diagnostic reasoning, management planning, and counseling. No single encounter or SP materially altered the study conclusions.

### Narrative feedback

Independent qualitative review of the narrative feedback generated by ChatGPT and Gemini found both systems produced detailed, accurate, and useful evaluations (Supplementary Tables S8–S11).

ChatGPT’s feedback emphasized communication structure and actionable recommendations for improvement. Reviewers noted that its feedback was highly organized and often included specific suggestions to improve transitions, diagnostic explanations, and patient counseling. However, the feedback was generally longer than Gemini’s and occasionally repetitive.

Gemini’s feedback emphasized diagnostic reasoning, scientific accuracy, and patient counseling. Reviewers described its feedback as concise, clinically focused, and easier to read, although it tended to be more checklist-oriented.

Claude and the human reviewer found that both LLMs consistently identified strengths and weaknesses in performance and generated feedback that was more detailed and comprehensive than typical feedback provided during standardized-patient encounters.

Overall, both LLMs generated narrative feedback that was judged to be accurate, comprehensive, and useful.

## Discussion

This study evaluated the ability of two large language models, ChatGPT and Gemini, to assess transcribed standardized physician-patient verbal interactions using a structured gold standard rubric composed of evaluation of history-taking (history-taking elements) and assessment and plan (presentation elements). Three principal findings emerged.

First, both LLMs demonstrated strong agreement with the human rater and each other in the assessment of history-taking elements. Mean scores were nearly identical across raters, and agreement was high across all rater pairs. Cohen’s kappa values ranged from 0.84–0.89, indicating close to perfect agreement in binary element ratings, while ICC values exceeded 0.90, indicating excellent reliability in scoring. These findings suggest that LLMs can accurately determine whether specific clinical information was obtained during a patient encounter when evaluation criteria are explicit and objective.

Second, the LLMs had lower agreement with each other and the human rater when assessing presentation elements, which assessed diagnostic reasoning, management planning, and patient counseling. Although overall scores remained similar across raters, kappa and ICC values were consistently lower than those observed for history-taking tasks. This result is not unexpected. Assessment of clinical reasoning and treatment planning requires greater interpretation than determining whether a specific history-taking element was elicited. Even when guided by gold standard rubrics, evaluation of assessment-and-plan components inherently involves subjective judgment (Haring et al., 2020; Daniel et al., 2019). The confidence intervals for presentation-element ICCs were also substantially wider, particularly for ChatGPT-human agreement (ICC 0.61, 95% CI 0.13–0.86), suggesting greater variability and uncertainty regarding the true level of agreement in this more interpretive assessment domain (Mokkink and Eekhout, 2026; Wilms et al., 2020).

Third, both LLMs generated qualitative narrative feedback that all reviewers considered detailed, accurate, and clinically useful. Although the two models differed in style, both consistently identified strengths and deficiencies in student performance. ChatGPT tended to emphasize structure and actionable recommendations, whereas Gemini focused more heavily on clinical reasoning, scientific accuracy, and patient counseling. These findings extend our prior work demonstrating the effectiveness of LLM-generated feedback for written clinical documentation and suggest that similar benefits may be achievable for transcribed verbal clinical interactions.

The observed difference between objective and interpretive evaluation tasks has important implications for future applications of LLM-based assessment. Performance was strongest when assessment criteria were explicit and directly observable. As evaluation tasks become more dependent on clinical judgment, reliability decreases, although overall agreement remained acceptable. Future studies should examine whether additional prompting strategies, consensus methods, or specialty-specific rubrics can improve reliability for more subjective assessment domains.

This study has several strengths. The use of a reliable clinical scenario, standardized patients, a gold standard rubric, and independently verified transcripts created a controlled environment in which LLM performance could be rigorously assessed. The study also evaluated both quantitative scoring and qualitative feedback generation, providing a more comprehensive assessment of LLM utility.

Several limitations should be acknowledged. We used a standardized patient–medical student paradigm, a well-established model of physician–patient verbal interactions that provides a controlled setting in which to evaluate LLM assessment and feedback (Wang et al., 2025; Jani et al., 2020; Epstein, 2007; Landis, 1977). The study was conducted at a single institution, involved only 12 encounters and one clinical condition, and may not generalize to other clinical scenarios, clinicians, healthcare settings, or real-world encounters. The leave-one-out sensitivity analyses did not materially alter the overall pattern of agreement, but the small sample size limited precision, particularly for presentation-element ICCs, which had wide confidence intervals. The evaluation using transcript-based review rather than direct assessment of audio recordings excluded paralanguage. Another limitation is that we assessed the presence or absence of information and not the quality with which information was elicited, communicated, or explained. The LLMs’ narrative feedback was evaluated qualitatively without a standardized quantitative rating scale or formal measures of inter-rater reliability. In addition, ChatGPT and Gemini participated in the evaluation of narrative feedback generated by their respective models, introducing potential self-evaluation bias and limiting the independence of the narrative feedback assessment.

In conclusion, ChatGPT and Gemini accurately evaluated transcribed standardized physician–patient verbal interactions in an SP-medical student paradigm and generated detailed, high-quality narrative feedback. Agreement was strongest for objective information-acquisition evaluation tasks and lower for more interpretive assessment-and-plan evaluation tasks. LLM-based assessment systems may provide consistent, efficient, and objective evaluation of clinical communication. These capabilities may support quality improvement initiatives, research, and future guideline-based evaluation of physician-patient encounters.

## Data Availability

The original contributions presented in the study are included in the article/Supplementary material, further inquiries can be directed to the corresponding author.
