# Supplementary material for: Large language model evaluation and feedback of transcribed simulated physician-patient verbal interactions
Source: Front Artif Intell. 2026 Sep 21;9:1936749. doi: 10.3389/frai.2026.1936749 (PMC13635307; doi:10.3389/frai.2026.1936749)
Supplement: Supplementary file 1 [file Data_Sheet_1.docx]

**Supplementary Tables**

**Table S1. Percent of history-taking elements rated present**

| History-taking elements | Percent present per human rater | Percent present per ChatGPT | Percent present per Gemini |
| --- | --- | --- | --- |
| Chief complaint |  |  |  |
| The patient is here to discuss his chest discomfort. | 100% | 100% | 100% |
| The chest discomfort started 3 weeks ago | 100% | 83% | 92% |
| History of present illness |  |  |  |
| The chest discomfort is pressure-like | 100% | 100% | 100% |
| The chest discomfort sometimes feels like a burning sensation. | 100% | 100% | 100% |
| The chest discomfort is intermittent. | 100% | 83% | 100% |
| The chest discomfort is mostly brought on by physical exertion. | 100% | 100% | 100% |
| Sometimes large meals bring the chest discomfort on. | 100% | 92% | 92% |
| The chest discomfort has improved with resting. | 100% | 100% | 100% |
| Antacids can provide some relief for the chest discomfort, but only sometimes. | 92% | 92% | 92% |
| It is hard for the patient to breathe when he is having the chest discomfort. | 91% | 91% | 91% |
| The chest discomfort has become more frequent and intense over time. | 83% | 100% | 100% |
| The chest discomfort radiates to the left neck and occasionally to the upper abdomen. | 83% | 67% | 83% |
| The chest discomfort was 4/10 when it first started, but lately it’s been up to 8/10 during episodes. | 75% | 75% | 75% |
| The patient has had similar pain occasionally over the past year but never this severe. | 75% | 75% | 83% |
| Now the chest discomfort is sometimes happening even at rest. | 70% | 70% | 70% |
| The chest discomfort is in the center of his chest. | 67% | 58% | 58% |
| The chest discomfort is brought on by emotional stress. | 58% | 58% | 58% |
| The chest discomfort first began while the patient was walking uphill. | 42% | 42% | 42% |
| Review of Systems |  |  |  |
| The patient has lightheadedness during episodes. | 75% | 75% | 67% |
| The patient has not had racing heartbeat or felt his heartbeat in his chest. | 75% | 75% | 67% |
| The patient has not had nausea or vomiting. | 67% | 67% | 67% |
| The patient has not had fever. | 58% | 75% | 58% |
| The patient occasionally has heart burn. | 45% | 45% | 36% |
| The patient has not had leg swelling. | 42% | 50% | 50% |
| The patient has sweating during episodes. | 33% | 33% | 33% |
| The patient has not had cough. | 25% | 25% | 25% |
| The patient has not had calf pain. | 17% | 8% | 8% |
| The patient has not had either tarry black stools or blood in the stools. | 8% | 8% | 8% |
| The patient has not had fatigue. | 0% | 0% | 0% |
| Past medical history, medications, and allergies |  |  |  |
| The patient was diagnosed with hypertension. | 100% | 100% | 92% |
| The patient was diagnosed with hyperlipidemia. | 100% | 100% | 100% |
| The patient is on 10 mg/day of lisinopril. | 92% | 83% | 83% |
| The patient is on 20 mg/day of atorvastatin. | 92% | 83% | 83% |
| The patient has taken over the counter antacids | 92% | 83% | 92% |
| The patient had an umbilical hernia repair years before. | 83% | 83% | 75% |
| The patient has not been hospitalized before except possibly for the hernia. | 67% | 58% | 67% |
| The patient takes no vitamins or herbs. | 58% | 67% | 58% |
| The patient has no drug allergies. | 58% | 58% | 58% |
| The patient misses some doses of lisinopril and atorvastatin. | 42% | 42% | 42% |
| The patient has no environmental allergies. | 42% | 42% | 50% |
| The patient has no food allergies. | 33% | 33% | 42% |
| The patient has no significant past injuries. | 17% | 42% | 25% |
| Family History |  |  |  |
| The patient’s father died from a heart attack at the age of 70. | 100% | 100% | 100% |
| The patient’s mother has diabetes. | 83% | 83% | 75% |
| The patient has 2 siblings who are alive and healthy. | 56% | 56% | 56% |
| Social history |  |  |  |
| The patient drinks 1-2 alcoholic beverages per week. | 90% | 90% | 90% |
| The patient has never used recreational drugs. | 75% | 67% | 67% |
| The patient’s diet consists of a lot of fruit and vegetables. | 67% | 58% | 50% |
| Until the chest discomfort, the patient exercised with regular walks. | 67% | 67% | 92% |
| The patient works as a chef. | 58% | 58% | 58% |
| The patient is stressed over his chest discomfort. | 58% | 50% | 67% |
| The patient is married. | 50% | 42% | 50% |
| The patient quit smoking 10 years ago. | 50% | 58% | 58% |
| The patient lives with his wife and child. | 27% | 27% | 27% |
| The patient is stressed over his work. | 25% | 25% | 25% |

**Table S2. Percent of presentation elements rated present**

| Presentation Elements | Percent present per human rater | Percent present per ChatGPT | Percent present per Gemini |
| --- | --- | --- | --- |
| Assessment |  |  |  |
| The student states a leading diagnosis. | 100% | 100% | 100% |
| The student identifies coronary artery disease or acute coronary syndrome as the leading diagnosis. | 100% | 100% | 92% |
| The student explains why a cardiac cause of chest pain is being considered. | 92% | 100% | 100% |
| The student provides a clear rationale for concern about ischemic heart disease. | 92% | 100% | 92% |
| The student mentions a gastrointestinal cause of chest pain as part of the differential. | 67% | 75% | 64% |
| The student explains potential consequences of heart disease (e.g., MI other cardiac complications). | 67% | 92% | 75% |
| Plan |  |  |  |
| The student states the need for further evaluation. | 100% | 83% | 100% |
| The student provides a patient-friendly explanation for recommended tests or treatments. | 100% | 92% | 100% |
| The plan includes an ECG (heart tracing) as part of the evaluation. | 67% | 58% | 73% |
| The plan includes appropriate cardiac imaging (e.g., echocardiogram, stress test, coronary CT, or catheterization). | 67% | 58% | 82% |
| The student recommends lifestyle modifications to reduce cardiovascular risk (e.g., blood pressure control, medication adherence). | 50% | 58% | 58% |
| The student discussed starting or changing at least one medication for angina (such as nitroglycerin, beta-blocker, antihypertensive, or improved medication adherence). | 42% | 50% | 58% |
| The plan includes cardiac biomarkers (e.g., troponin). | 33% | 42% | 42% |
| The plan includes treatment for or evaluation of reflux/GERD or another gastrointestinal cause. | 33% | 58% | 45% |
| The treatment plan includes aspirin. | 25% | 25% | 25% |
| The student discusses red-flag symptoms that warrant urgent medical attention (e.g., worsening chest pain, dyspnea, syncope). | 8% | 17% | 8% |
| Communication and closing |  |  |  |
| The student communicates appropriate concern while reassuring the patient. | 100% | 100% | 100% |
| The student asks if the patient has questions near the end of the visit. | 92% | 100% | 100% |
| The student explains or avoids medical terminology not likely familiar to patients (minor slips acceptable). | 83% | 100% | 100% |

**Table S3 Percent present scores by rater**

| Encounter | History-taking Element Score | | | Presentation Score | | |
| --- | --- | --- | --- | --- | --- | --- |
|  | Human | ChatGPT | Gemini | Human | ChatGPT | Gemini |
| Student 1 | 74% | 77% | 75% | 68% | 68% | 68% |
| Student 2 | 79% | 81% | 81% | 84% | 68% | 79% |
| Student 3 | 55% | 51% | 57% | 68% | 79% | 79% |
| Student 4 | 67% | 67% | 67% | 79% | 84% | 79% |
| Student 5 | 59% | 59% | 59% | 89% | 95% | 89% |
| Student 6 | 62% | 62% | 60% | 58% | 74% | 58% |
| Student 7 | 56% | 58% | 60% | 58% | 68% | 68% |
| Student 8 | 74% | 70% | 75% | 63% | 68% | 63% |
| Student 9 | 82% | 80% | 80% | 63% | 68% | 68% |
| Student 10 | 56% | 54% | 54% | 58% | 68% | 68% |
| Student 11 | 60% | 60% | 60% | 84% | 79% | 84% |
| Student 12 | 76% | 67% | 65% | 58% | 68% | 68% |
| Mean score (SD) | 67% (10%) | 65% (10%) | 65% (10%) | 69% (12%) | 73% (9%) | 73% (9%) |

Values may differ from Table 1 due to differences in calculation and rounding.

**Table S4 Leave-one-out sensitivity analysis of history-taking element Cohen's κ**

| Analysis Condition | ChatGPT-Gemini  (**κ**, 95% CI) | ChatGPT-Human (**κ**, 95% CI) | Gemini-Human  (**κ**, 95% CI) |
| --- | --- | --- | --- |
| Full dataset | 0.84 (0.80—0.89) | 0.89 (0.86—0.93) | 0.89 (0.86—0.93) |
| Excluding  Student 1 | 0.84 (0.80—0.89) | 0.89 (0.86—0.93) | 0.89 (0.85—0.93) |
| Excluding  Student 2 | 0.85 (0.80—0.89) | 0.89 (0.85—0.93) | 0.90 (0.86—0.93) |
| Excluding  Student 3 | 0.84 (0.78—0.88) | 0.88 (0.84—0.92) | 0.88 (0.84—0.92) |
| Excluding  Student 4 | 0.83 (0.78—0.88) | 0.88 (0.84—0.92) | 0.88 (0.84—0.92) |
| Excluding  Student 5 | 0.83 (0.79—0.88) | 0.89 (0.85—0.93) | 0.88 (0.84—0.92) |
| Excluding  Student 6 | 0.83 (0.79—0.88) | 0.89 (0.85—0.93) | 0.89 (0.85—0.93) |
| Excluding  Student 7 | 0.88 (0.84—0.92) | 0.89 (0.86—0.93) | 0.94 (0.91—0.97) |
| Excluding  Student 8 | 0.84 (0.80—0.89) | 0.90 (0.86—0.94) | 0.89 (0.85—0.93) |
| Excluding  Student 9 | 0.84 (0.79—0.88) | 0.89 (0.85—0.93) | 0.89 (0.85—0.93) |
| Excluding  Student 10 | 0.83 (0.79—0.88) | 0.89 (0.86—0.93) | 0.89 (0.85—0.93) |
| Excluding  Student 11 | 0.83 (0.79—0.88) | 0.89 (0.85—0.93) | 0.88 (0.84—0.92) |
| Excluding  Student 12 | 0.86 (0.83—0.91) | 0.92 (0.88—0.95) | 0.91 (0.88—0.95) |
| Excluding  SP1 (students 1—4) | 0.82 (0.77—0.88) | 0.88 (0.83—0.92) | 0.87 (0.82—0.92) |
| Excluding  SP2 (students 5—8) | 0.87 (0.82—0.92) | 0.89 (0.85—0.94) | 0.92 (0.88—0.96) |
| Excluding  SP3 (students 9—12) | 0.84 (0.78—0.89) | 0.92 (0.88—0.96) | 0.89 (0.85—0.94) |

**Table S5 Leave-one-out sensitivity analysis of history-taking element ICC**

| Analysis Condition | ChatGPT-Gemini  (ICC, 95% CI) | ChatGPT-Human (ICC, 95% CI) | Gemini-Human (ICC, 95% CI) |
| --- | --- | --- | --- |
| Full dataset | 0.97 (0.89—0.98) | 0.94 (0.82—0.98) | 0.93 (0.78—0.98) |
| Excluding Student 1 | 0.96 (0.88—0.99) | 0.94 (0.79—0.98) | 0.92 (0.75—0.98) |
| Excluding Student 2 | 0.95 (0.85—0.99) | 0.93 (0.77—0.98) | 0.91 (0.72—0.98) |
| Excluding Student 3 | 0.98 (0.92—0.99) | 0.94 (0.80—0.98) | 0.92 (0.74—0.98) |
| Excluding Student 4 | 0.97 (0.88—0.99) | 0.94 (0.81—0.98) | 0.93 (0.76—0.98) |
| Excluding Student 5 | 0.96 (0.88—0.99) | 0.94 (0.80—0.98) | 0.92 (0.75—0.98) |
| Excluding Student 6 | 0.97 (0.89—0.99) | 0.94 (0.81—0.98) | 0.93 (0.76—0.98) |
| Excluding Student 7 | 0.97 (0.89—0.99) | 0.94 (0.79—0.98) | 0.93 (0.77—0.98) |
| Excluding Student 8 | 0.98 (0.91—0.99) | 0.95 (0.82—0.99) | 0.92 (0.75—0.98) |
| Excluding Student 9 | 0.96 (0.85—0.99) | 0.93 (0.77—0.98) | 0.91 (0.70—0.97) |
| Excluding Student 10 | 0.96 (0.86—0.99) | 0.94 (0.79—0.98) | 0.92 (0.73—0.98) |
| Excluding Student 11 | 0.96 (0.88—0.99) | 0.94 (0.80—0.98) | 0.93 (0.75—0.98) |
| Excluding Student 12 | 0.97 (0.89—0.99) | 0.94 (0.82—0.98) | 0.98 (0.94—1.00) |
| Excluding SP1 (students 1—4) | 0.97 (0.86—0.99) | 0.92 (0.67—0.98) | 0.90 (0.61—0.98) |
| Excluding SP2 (students 5—8) | 0.98 (0.89—1.00) | 0.94 (0.76—0.99) | 0.92 (0.69—0.98) |
| Excluding SP3 (students 9—12) | 0.95 (0.80—0.99) | 0.97 (0.84—0.99) | 0.98 (0.89—0.99) |

**Table S6 Leave-one-out sensitivity analysis of presentation-element Cohen's κ**

|  | ChatGPT-Gemini  (**κ**, 95% CI) | ChatGPT-Human  (**κ**, 95% CI) | Gemini-Human (**κ**, 95% CI) |
| --- | --- | --- | --- |
| Full dataset | 0.72 (0.62—0.82) | 0.62 (0.51—0.74) | 0.64 (0.53—0.75) |
| Excluding Student 1 | 0.63 (0.52—0.75) | 0.65 (0.54—0.76) | 0.72 (0.61—0.82) |
| Excluding Student 2 | 0.67 (0.57—0.78) | 0.65 (0.53—0.76) | 0.77 (0.67—0.87) |
| Excluding Student 3 | 0.61 (0.50—0.73) | 0.63 (0.51—0.75) | 0.70 (0.59—0.81) |
| Excluding Student 4 | 0.62 (0.50—0.74) | 0.65 (0.53—0.76) | 0.70 (0.59—0.81) |
| Excluding Student 5 | 0.62 (0.50—0.73) | 0.62 (0.50—0.73) | 0.72 (0.61—0.82) |
| Excluding Student 6 | 0.64 (0.53—0.76) | 0.64 (0.53—0.76) | 0.73 (0.62—0.83) |
| Excluding Student 7 | 0.63 (0.51—0.75) | 0.65 (0.53—0.76) | 0.72 (0.61—0.82) |
| Excluding Student 8 | 0.62 (0.50—0.74) | 0.65 (0.53—0.76) | 0.70 (0.59—0.81) |
| Excluding Student 9 | 0.60 (0.47—0.72) | 0.64 (0.52—0.75) | 0.72 (0.61—0.82) |
| Excluding Student 10 | 0.63 (0.51—0.75) | 0.62 (0.50—0.74) | 0.72 (0.61—0.82) |
| Excluding Student 11 | 0.61 (0.49—0.73) | 0.64 (0.52—0.75) | 0.71 (0.60—0.82) |
| Excluding Student 12 | 0.61 (0.48—0.73) | 0.65 (0.53—0.76) | 0.75 (0.65—0.85) |
| Excluding SP1 (students 1—4) | 0.67 (0.55—0.80) | 0.65 (0.52—0.78) | 0.74 (0.62—0.86) |
| Excluding SP2 (students 5—8) | 0.64 (0.51—0.78) | 0.64 (0.50—0.78) | 0.70 (0.56—0.83) |
| Excluding SP3 (students 9—12) | 0.55 (0.40—0.70) | 0.63 (0.49—0.76) | 0.72 (0.60—0.85) |

**Table S7 Leave-one-out sensitivity analysis of presentation-element ICC**

|  | ChatGPT-Gemini (ICC, 95% CI) | ChatGPT-Human (ICC, 95% CI) | Gemini-Human (ICC, 95% CI) |
| --- | --- | --- | --- |
| Full dataset | 0.74 (0.34—0.92) | 0.61 (0.13—0.86) | 0.83 (0.47—0.94) |
| Excluding Student 1 | 0.74 (0.29—0.92) | 0.61 (0.17—0.91) | 0.83 (0.43—0.95) |
| Excluding Student 2 | 0.81 (0.45—0.94) | 0.70 (-0.03—0.92) | 0.82 (0.31—0.95) |
| Excluding Student 3 | 0.74 (0.29—0.92) | 0.65 (0.15—0.89) | 0.87 (0.58—0.96) |
| Excluding Student 4 | 0.74 (0.27—0.92) | 0.59 (0.07—0.86) | 0.82 (0.48—0.97) |
| Excluding Student 5 | 0.57 (-0.02—0.87) | 0.41 (-0.13—0.78) | 0.77 (0.30—0.93) |
| Excluding Student 6 | 0.86 (0.56—0.96) | 0.68 (0.21—0.90) | 0.80 (0.37—0.95) |
| Excluding Student 7 | 0.74 (0.29—0.92) | 0.62 (0.11—0.88) | 0.86 (0.61—0.97) |
| Excluding Student 8 | 0.74 (0.28—0.92) | 0.61 (0.18—0.91) | 0.82 (0.41—0.95) |
| Excluding Student 9 | 0.74 (0.29—0.92) | 0.61 (0.18—0.91) | 0.83 (0.48—0.95) |
| Excluding Student 10 | 0.74 (0.29—0.92) | 0.62 (0.11—0.88) | 0.86 (0.55—0.96) |
| Excluding Student 11 | 0.74 (0.30—0.92) | 0.59 (0.06—0.87) | 0.80 (0.37—0.94) |
| Excluding Student 12 | 0.75 (0.34—0.92) | 0.62 (0.11—0.88) | 0.86 (0.61—0.97) |
| Excluding SP1 (students 1—4) | 0.79 (0.32—0.95) | 0.72 (0.03—0.94) | 0.86 (0.28—0.96) |
| Excluding SP2 (students 5—8) | 0.77 (0.23—0.94) | 0.48 (-0.27—0.86) | 0.73 (0.17—0.92) |
| Excluding SP3 (students 9—12) | 0.72 (0.15—0.93) | 0.59 (-0.04—0.89) | 0.87 (0.54—0.97) |

**Supplement S8: ChatGPT and Gemini’s narrative feedback to student 1**

| Student | ChatGPT | Gemini |
| --- | --- | --- |
| 1 | **Information Acquisition Feedback Narrative**  The medical student covered the majority of the gold standard elements in the interview. However, the following elements were **not addressed or were missing** from the interaction:   1. **Trigger Details:**    - The chest discomfort beginning while the patient was walking uphill was not elicited.    - Emotional stress as a trigger was not explored. 2. **Symptom Qualifiers:**    - Sweating and lightheadedness during episodes were not asked about.    - Palpitations or awareness of heartbeat were not addressed.    - Cough, calf pain, and fatigue were not covered. 3. **Adherence & Lifestyle:**    - Whether the patient misses doses of lisinopril and atorvastatin was not discussed.    - Stress related to work and to the chest discomfort itself was not explicitly explored.    - No mention was made of the patient’s prior exercise routine before symptoms began.   These gaps suggest the need for more comprehensive history-taking, particularly with respect to associated symptoms, emotional and lifestyle triggers, and medication adherence. Doing so can significantly impact diagnostic accuracy and the development of an appropriate management plan.  **Output Narrative Feedback**  The student demonstrated a competent and reassuring manner in delivering the assessment and plan, with a clear focus on patient education and empathy. They accurately synthesized many key historical elements into their diagnostic reasoning and communicated a reasonable and appropriate preliminary diagnosis of **angina**. The explanation of how exertion can lead to myocardial oxygen demand exceeding supply was well articulated in accessible language. The use of analogies and linking symptoms to physiological mechanisms was effective and helped contextualize the diagnosis for the patient.  The student provided a general overview of treatment options, mentioning **aspirin**, **lipid and blood pressure monitoring**, and **isosorbide mononitrate** for symptomatic relief. These suggestions were appropriate and addressed the underlying pathology. However, the explanation of the treatment plan could have benefited from more structure and specificity. For example, the student did not summarize the major supporting findings for angina clearly before launching into the pharmacologic options. This lack of recap limited the opportunity to reinforce the patient's understanding and validate the diagnostic reasoning.  Counseling was empathetic, with appropriate caution around worsening symptoms (“come straight to the emergency department”), but could have been more proactive in addressing lifestyle modification (e.g., exercise resumption, dietary adjustments, stress management). Furthermore, although the student mentioned "watching your heart" and listening to symptoms, more targeted counseling on medication adherence and risk factor modification (e.g., smoking history, hyperlipidemia, hypertension) would have enhanced the counseling component.  The student’s organization of information was generally adequate but lacked clear transitions between diagnosis, explanation, plan, and counseling. More explicit structuring (e.g., “First, let me explain what I think is going on. Then we’ll talk about what we can do and how you can take care of this at home.”) would improve clarity and patient engagement.  In summary, the student presented a reasonable and compassionate assessment and plan with appropriate medical reasoning, but greater structure, clearer summarization, and more comprehensive lifestyle counseling would strengthen future encounters. | **Information Acquisition Feedback Narrative**  The student performed a thorough history; however, several key diagnostic and social elements were missed:   - **Associated Symptoms:** The student failed to ask about **sweating (diaphoresis)**, **lightheadedness**, or **palpitations (racing heartbeat)** during chest pain episodes. These are critical for assessing cardiac distress. - **Pertinent Negatives:** While the student asked about GI symptoms, they did not screen for **cough**, **fatigue**, or **calf pain**. - **Triggers:** The student identified physical exertion and meals as triggers but did not explore **emotional stress** as a potential cause. - **Medication Adherence and Supplements:** The student confirmed the names and doses of medications but did not ask if the patient **misses any doses** or if they take **vitamins or herbs**. - **Past History:** The student did not confirm the absence of **past injuries**. - **Psychosocial Stressors:** Although the student discussed the patient's job as a chef, they did not inquire about **work-related stress** or the patient's **stress regarding the chest discomfort** itself.   **Output Narrative Feedback**  **Organization and Synthesis of Information**  The student demonstrated a strong ability to organize the acquired information into a coherent summary before transitioning to the diagnosis. By recapping the symptoms—specifically the 3-week duration, the location in the center of the chest, radiation to the neck/stomach, and the relationship with exertion—the student effectively synthesized the history to support their clinical reasoning. This summary served as an excellent bridge between the history-taking and the assessment phase.  **Presentation of Diagnosis**  The student provided a clear and accurate diagnosis of **angina**. The explanation of the pathophysiology was handled with a touch of wit and clarity, using the analogy of the heart "telling you" it needs rest. They correctly identified the mechanism as an imbalance between blood supply and demand during physical activity. However, while "angina" was mentioned, the student could have explicitly categorized it as "stable angina" to distinguish it from more acute presentations, though they did provide appropriate "red flag" warnings.  **Diagnostic and Therapeutic Plan**  The student articulated a multifaceted plan that included:   - **Pharmacotherapy**: Introducing aspirin and a nitrate (isosorbide mononitrate) for symptom management. - **Monitoring**: Proposing a re-evaluation of lipids and blood pressure. - **Administration**: Properly explaining how to use the nitrate "under the tongue" for acute relief.   The plan was logical and directly addressed the patient's existing conditions of hypertension and hyperlipidemia.  **Patient Counseling**  Counseling was a highlight of the interaction. The student effectively used **Safety Netting** by instructing the patient to go to the emergency department if the pain occurs at rest or worsens suddenly. They also advised the patient to "take it a little easier" in the short term. To improve, the student could have more explicitly linked the patient’s history of smoking (20 pack-years) to the current diagnosis during the counseling phase to reinforce lifestyle modifications. |

**Supplement S9: ChatGPT’s review of ChatGPT and Gemini’s narrative feedback to student 1**

**1. Overall Comparison of ChatGPT vs Gemini rater Feedback**

Both raters identify very similar missing diagnostic elements, indicating strong agreement about the student’s information-gathering gaps.

**Shared omissions identified**

Both raters note that the student failed to ask about:

- Sweating (diaphoresis), Lightheadedness, Palpitations, Cough, Calf pain, Fatigue, Emotional stress as a trigger, medication adherence, and Psychosocial/work stress

Suggesting high content agreement between raters and similar expectations for a cardiac chest pain review of systems

**ChatGPT noted two additional missing details:** Onset trigger: walking uphill, Prior exercise routine before symptoms

Gemini’s assessment of information acquisition is slightly more granular.

**2. Differences in Feedback Style and Focus**

**A. Organization and Structure**

**ChatGPT**

Highly structured evaluation:

- Strengths, Areas for improvement, Conclusion
- ChatGPT explicitly critiques transition structure between diagnosis, plan, and counseling.

**Gemini**

Narrative-heavy evaluation:

- Less structured segmentation, Some redundancy

**3. Differences in Clinical Reasoning Feedback**

**Gemini emphasis**

Gemini strongly praises the student's information synthesis:

“The student demonstrated a strong ability to organize the acquired information into a coherent summary.”

Gemini repeatedly highlights:

- clinical synthesis, narrative recap, reasoning linking symptoms to angina

**4. Counseling and Patient Communication**

Both raters agree the student demonstrated:

- Good empathy, Effective explanation of angina, Appropriate safety-netting

Examples cited:

- Emergency department warning for worsening pain, Clear explanation of supply–demand mismatch

Both raters also recommend more lifestyle counseling, including:

- Smoking risk discussion, Risk factor modification, Stress management

Agreement level here is very high.

**5. Evaluation of Gemini’s Evaluation Quality**

**Strengths of Gemini**

**1. Clinically accurate critique**

Gemini correctly identifies key omissions relevant to cardiac chest pain evaluation:

- autonomic symptoms, thromboembolic symptoms, psychosocial context, medication adherence

**2. Strong explanation of clinical reasoning**

Gemini explains why the diagnosis of angina makes sense, including:

- supply–demand mismatch, exertional symptoms, risk factors

**3. Good attention to patient communication**

Gemini appropriately highlights:

- rapport building, safety netting, clear explanations

**Weaknesses of Gemini**

**1. Less actionable improvement guidance**

ChatGPT provides clearer behavioral suggestions, such as:

- using structured transitions, summarizing findings before plan, linking risk factors to counseling

Gemini’s suggestions are more general (e.g., “more exhaustive review”).

**6. Overall Judgment of Gemini**

Overall rating of Gemini as an evaluator: Clinical accuracy is strong, there is frequent agreement with ChatGPT, the educational clarity and actionable feedback is moderately strong.

- Clinical accuracy: Strong
- Agreement with gold-standard elements: High
- Educational clarity: Moderate
- Actionable feedback: Moderate

**Final evaluation**

Gemini provides clinically accurate and largely consistent feedback, but the evaluation is:

- less structured and less actionable

Compared with ChatGPT, it is pedagogically weaker but clinically sound.

- Both raters identify almost identical clinical omissions.
- ChatGPT provides more structured, precise, and actionable feedback.

**Supplement S10: Gemini’s review of ChatGPT and Gemini’s narrative feedback for student 1**

**Comparison of ChatGPT and Gemini Feedback**

Both raters provide a structured assessment of the student’s performance across information acquisition, clinical reasoning, and communication. They are in high agreement regarding the student's clinical hits and misses.

**1. Areas of Agreement (Clinical Content)**

- **Strengths identified by both:**
  - Effective synthesis of history into a clear diagnosis of angina.
  - Successful use of the "supply and demand" pathophysiology analogy.
  - Appropriate pharmacologic plan (aspirin, nitrates) and "safety netting" (ER instructions).
  - Strong rapport and empathy.
- **Omissions identified by both:**
  - Failure to screen for diaphoresis (sweating) and lightheadedness.
  - Missing the assessment of medication adherence (specifically missing doses).
  - Failure to explore emotional stress as a trigger or a psychosocial factor.
  - Omission of "pertinent negatives" like cough and calf pain.

**2. Divergent Focus and Nuance**

- **ChatGPT (ChatGPT):** Focuses more on procedural flow. It critiques the student’s lack of "clear transitions" between the diagnosis, plan, and counseling.
- **Gemini (Gemini):** Focuses more on clinical precision. It suggests the student should have explicitly categorized the condition as "stable angina" to distinguish it from acute presentations. It also highlights the student's missed opportunity to link the patient's smoking history to the current diagnosis.

**Evaluation of ChatGPT’s (ChatGPT) Feedback**

ChatGPT provides a highly professional, balanced, and actionable evaluation. Below is an assessment of its quality:

**Strengths of ChatGPT’s Evaluation**

- **Focus on Communication Structure:** ChatGPT identifies a "soft skill" gap that Gemini largely overlooks—the importance of signposting. By suggesting phrases like *"First, let me explain..."*, it provides the student with a concrete tool to improve patient engagement.
- **Comprehensive Categorization:** The feedback is logically divided into "Strengths" and "Areas for Improvement," making it easy for a student to digest and prioritize.
- **Holistic Perspective:** It notes the lack of inquiry into the patient’s prior exercise routine, a detail that helps establish a clinical baseline which Gemini did not emphasize.

**Weaknesses of ChatGPT’s Evaluation**

- **Less Clinical Nuance:** While ChatGPT identifies that the student diagnosed "angina," it does not offer the higher-level clinical critique found in Gemini regarding the specific classification of "stable" vs. "unstable".

**Conclusion on ChatGPT**

ChatGPT is an excellent coach for structure and rapport. It excels at helping the student understand *how* to conduct an interview. While slightly less focused on the fine-grained medical classification than Gemini, its emphasis on transition statements and specific scoring makes it a very effective tool for student development.

**Supplement S11: Claude’s review of ChatGPT and Gemini’s narrative feedback for student 1**

Information acquisition — both raters agreed on most items missed. There were a few discrepancies

Clinical Output

Raters agreed on most ratings, but they had different emphasis

ChatGPT was critical of:

- Lack of clear transitions between diagnosis, plan, and counselling sections.
- Did not summarise key findings before launching into pharmacologic options.
- Organisation was "generally adequate" but needed more explicit structuring.

ChatGPT’s Tone

- Constructive but highlights structural weaknesses prominently.

Gemini

Praised highly

- Recap of symptoms before diagnosis was called "excellent bridge" to the assessment.
- Sublingual nitrate instructions were explicitly commended.
- Using the patient's career as a chef to build rapport was highlighted positively.

Suggested upgrade

- Should have labelled diagnosis as "stable angina" specifically, not just "angina."

Overall verdict

ChatGPT: Solid but developing. Emphasises need to refine history-taking completeness, improve structural organisation, and expand depth of patient counselling.

Gemini:

Strongest attribute is clinical synthesis and reasoning. Encourages more exhaustive cardiac review of systems and deeper lifestyle exploration as the path to expert performance.

Here's a summary of the key differences and agreements between the two raters:

**Where they fully agree:** Both raters flagged the same core omissions — diaphoresis, lightheadedness, palpitations, emotional stress as a trigger, medication adherence, and psychosocial stress. They also agreed that the diagnosis, safety netting, and rapport were strengths, while smoking history was underused in counselling.

**Where ChatGPT (ChatGPT) was more critical:** It focused on the *structural organisation* of the output — the student launched into pharmacologic options without first summarising findings, and transitions between sections were abrupt. ChatGPT seemed to penalise this more explicitly.

**Where Gemini (Gemini) was more generous:** It actively praised the symptom recap before diagnosis as an "excellent bridge," commended the sublingual nitrate instructions, and noted the use of the patient's chef career as a rapport-building strength — none of which ChatGPT mentioned. Gemini's one notable unique critique was that the student should have specified "stable angina" rather than just "angina."

**Bottom line:** Both raters see a competent, empathetic student with good clinical reasoning but an incomplete review of systems. The disagreement is mainly one of framing — ChatGPT foregrounds structural weaknesses in the output, while Gemini foregrounds synthesis strengths.
